# Spore Germination of the Obligate Biotroph *Spongospora subterranea*: Transcriptome Analysis Reveals Germination Associated Genes

**DOI:** 10.3389/fmicb.2021.691877

**Published:** 2021-06-16

**Authors:** Sadegh Balotf, Robert S. Tegg, David S. Nichols, Calum R. Wilson

**Affiliations:** ^1^Tasmanian Institute of Agriculture, New Town Research Laboratories, University of Tasmania, New Town, TAS, Australia; ^2^Central Science Laboratory, University of Tasmania, Hobart, TAS, Australia

**Keywords:** obligate biotrophic, spore germination, *Spongospora subterranea*, powdery scab, transcriptomics

## Abstract

For soilborne pathogens, germination of the resting or dormant propagule that enables persistence within the soil environment is a key point in pathogenesis. *Spongospora subterranea* is an obligate soilborne protozoan that infects the roots and tubers of potato causing root and powdery scab disease for which there are currently no effective controls. A better understanding of the molecular basis of resting spore germination of *S. subterranea* could be important for development of novel disease interventions. However, as an obligate biotroph and soil dwelling organism, the application of new omics techniques for the study of the pre-infection process in *S. subterranea* has been problematic. Here, RNA sequencing was used to analyse the reprogramming of *S. subterranea* resting spores during the transition to zoospores in an *in-vitro* model. More than 63 million mean high-quality reads per sample were generated from the resting and germinating spores. By using a combination of reference-based and *de novo* transcriptome assembly, 6,664 unigenes were identified. The identified unigenes were subsequently annotated based on known proteins using BLAST search. Of 5,448 annotated genes, 570 genes were identified to be differentially expressed during the germination of *S. subterranea* resting spores, with most of the significant genes belonging to transcription and translation, amino acids biosynthesis, transport, energy metabolic processes, fatty acid metabolism, stress response and DNA repair. The datasets generated in this study provide a basic knowledge of the physiological processes associated with spore germination and will facilitate functional predictions of novel genes in *S. subterranea* and other plasmodiophorids. We introduce several candidate genes related to the germination of an obligate biotrophic soilborne pathogen which could be applied to the development of antimicrobial agents for soil inoculum management.

## Introduction

Our understanding of the pre-infection process in plant-pathogen interaction events has rapidly increased in the last 20 years with the advent of new molecular tools. However, with biotrophic soil dwelling pathogens, the difficulty of obtaining high-quality DNA, RNA and protein has made the application of new omics technologies difficult (Singh et al., 2018). The plasmodiophorid pathogen *Spongospora subterranea* f. sp. *subterranea* (Wallroth) Lagerheim falls within this category with a complex obligate biotrophic lifestyle associated with the roots and tubers of potato. Root infection by *S. subterranea* results in root disfunction and reduced capacity for water and nutrients absorption, causing reductions in plant productivity and yield (Falloon et al., 2015). Root galls can also form approximately 1–3 months following the first zoospore-mediated root infection. New resting spores of *S. subterranea* are produced inside the galls and liberate into the soil at the end of the pathogen life cycle. The pathogen also infects developing tubers resulting in the disease powdery scab, another source of resting spores, which are expressed as unsightly lesions (Wilson, 2016). These reduce the quality of tubers for fresh market sale, increase processing costs of potato products such as fries and chips and downgrade the value of seed potatoes, as diseased tubers may fail certification standards (Balendres et al., 2016b). Despite the importance of this pathogen, *S. subterranea* has received relatively little research attention.

For soilborne pathogens, the first step in pathogenesis is the germination of dormant spores, a process often associated with root exudation of phytochemicals from a host plant in the near vicinity. However, in some instances germination can be triggered by alternate compounds in the absence of a host (Pinto et al., 2020). Resting spore germination in *S. subterranea* is a complex and poorly understood process (Balendres, 2017). The resting spores are present as aggregates of several hundreds of individual spores, termed sporosori. Within a sporosorus, not all individual resting spores at any one time will be stimulated to germinate by incubation with stimulants such as Hoagland’s solution; a proportion of resting spores will remain constitutively dormant (Balendres et al., 2017). In *Plasmodiophora brassicae* Voronin, one of the most closely related plasmodiophorid to *S. subterranea*, both dormant and non-dormant resting spores exist in the population. The dormant spores require an external germination stimulant such as the host root exudate or nutrient solution. After germination, zoospores also need water to move toward the host roots through the soil. Germination of a small proportion of resting spores is shown to be sufficient to initiate root infection (Neuhauser et al., 2010).

The molecular mechanisms of the interactions between plasmodiophorids and their hosts have been investigated using RNA sequencing (RNA-seq) technology (Jia et al., 2017; Dakouri et al., 2018; Irani et al., 2018; Galindo-González et al., 2020). Schwelm et al. (2015) reported the genome draft of *P. brassicae* from genomic DNA of resting spores from the single spore isolate whilst the transcriptome of *S. subterranea* was obtained from resting spores in potato root galls. In total, 9,730 and 12,732 (7,490 were full-length) proteins were predicted for *P. brassicae* and *S. subterranea*, respectively (Schwelm et al., 2015). Lekota et al. (2019) analysed the responses of susceptible and resistant potato cultivars to *S. subterranea* infection and identified several defence−response genes that showed contrasting expression patterns between the susceptible and resistant cultivars. There are several other examples of the interaction between plasmodiophorids and their hosts (Barr et al., 1995; Zhao et al., 2017; Galindo-González et al., 2020; Ji et al., 2021). However, most of these studies focused on the post-infection phase of the host-pathogen interaction (in planta analysis) while in contrast there is insufficient information available on critical pre-infection events including resting spore germination processes in plasmodiophorids. This can be explained by the non-culturable and plant-associated nature of these pathogens. We previously reported a density gradient centrifugation using the Ludox^®^ technique for purification of *S. subterranea* resting spores (Balotf et al., 2020). In the present study, RNA-seq analysis was employed to gain insight into the transcriptional reprogramming that occurs during the germination of *S. subterranea* resting spores. This provides the first transcriptome analysis of *S. subterranea* during the germination of resting spores.

## Materials and Methods

### Sample Collection and Purification

*Spongospora subterranea* spores were scraped and purified from powdery scab-infected potato tubers, collected from fields in Devonport, Tasmania, Australia as previously described (Balotf et al., 2020). Briefly, the heavily infected tubers were washed for 2–3 min using tap water and left to dry for 3 days (air dried). The powdery scab lesions were excised from the tuber and dried for 72 h at 40°C. Then 100 mg of the dried lesions were gently homogenised in 3 mL sterile water using a pestle and mortar. Large debris was removed from the suspension by filtration through two layers of cheesecloth. The spores were further purified through a Ludox^®^ (HS-40 colloidal silica, Sigma, NSW, Australia) gradient centrifugation (4200 × *g*, 15 min) (Balotf et al., 2020). After washing with sterile water, the spore purity was examined microscopically (200–400 X). To confirm the presence of *S. subterranea* in the purified samples, a PCR was performed with primer pairs targeted to the 18S rRNA gene (Balotf et al., 2021).

### Spore Germination

The purified spores were divided into two parts. The first part was used for the germinating spore treatment. Spores were suspended in Hoagland’s solution, a known stimulant of resting spore germination (Balendres et al., 2016a), and incubated at 25°C for 3 days. Resting spore germination was verified by observation of active zoospores by light microscopy (200–400 X) within a 10 μl sample of the solution. The samples were then centrifuged at 10,000 *g* for 1 min and the supernatant discarded. The second part was incubated at 25°C as dry sporosori for 3 days and served as the non-germinated spores treatment. Each treatment included three independent biological replications.

### RNA Preparation for Sequencing

Total RNA was extracted from 50 mg of each sample using the RNeasy Plant Mini Kit (Qiagen, Hilden, Germany) following the manufacturer’s protocol. Any contaminating genomic DNA was removed from the extracted RNA using the RNase-free DNase Set following the manufacturer’s protocol (Qiagen, Hilden, Germany). The yield of the RNA was measured with a Qubit fluorometer using the Qubit RNA Broad-Range kit (Invitrogen, Waltham, MA, United States). RNA quality was assessed by a 2100 Bioanalyser RNA Nano Chip (Agilent, Palo Alto, CA, United States). One μg of RNA from each sample was used for further analysis. Library construction was generated using the Illumina TruSeq^TM^ Stranded mRNA Library Preparation Kit TruSeq (Illumina, San Diego, CA, United States) and then sequenced on a NovaSeq 6000 instrument at Macrogen, Inc. (Macrogen, Seoul, South Korea) to obtain ∼100 million 2 × 150 bp reads/sample.

### Transcriptome Assembly

The RNA-seq analysis was performed using next-generation sequencing (NGS) analysis tools available on the Galaxy platform (Afgan et al., 2018). Specifically, the NGS raw data were processed using the *de novo* transcript reconstruction protocol described by Freeberg and Heydarian (2020). The quality of raw reads was assessed with FastQC 0.11.9 tools. The Trimmomatic tool was used to trim the sequence reads by removing the remaining TrueSeq Illumina adaptors in the reads. This program also discards unpaired reads from paired-end RNA-seq output. After the removal of low-quality reads, the read quality was assessed again using FastQC. Forward and reverse reads were mapped to the *S. subterranea* draft genome (Ciaghi et al., 2018) to remove the non-target reads using the HISAT2 2.1.0 tool with a minimum fragment length of 20. The Stringtie 2.1.1 tool was used for *de novo* transcriptome reconstruction of the assembled reads. A transcriptome database was created using the tool Stringtie-Merge by combining redundant transcript structures across the six assembled samples (the Stringtie outputs). Finally, GFFcompare 0.11.2, FeatureCounts 1.6.4 and DESeq 2.11.40.6 tools were used to compare and visualize the relative expression abundances between the two groups (non-germinating and germinating spores) (Love et al., 2014). The transcript’s abundance was calculated using the fragments per kilobase of per million mapped reads (FPKM). A false discovery rate (FDR) threshold of 5% was used to determine DEGs. The workflow for the RNA-seq experiment is provided in Supplementary Figure 1.

### Functional Annotation and Gene Ontology

To provide comprehensive annotation for the final unigenes, the transcripts were subject to BLAST analysis against the *S. subterranea* database in UniProt^1^ (containing 11,129 proteins), NCBI non-redundant database and the Swissprot database with an e-value cut-off of 1 × 10^––5^. Gene ontology (GO) terms (describing the biological process and molecular function) and the gene pathway networks for the DEGs were derived from DAVID^2^, UniProt, the Kyoto Encyclopedia of Genes and Genomes (KEGG) entries^3^ and STRING.^4^ Enzyme Commission (EC) was obtained from the Expasy database.^5^ The heatmap was drawn using Perseus software (v. 1.5.0.15)^6^ and the PCA plot was obtained from the Galaxy platform.

### RNA-Seq Validation With Real-Time PCR

To validate the RNA-Seq data, 10 randomly selected DEGs (up and downregulated, 5 each) were subject to quantitative real-time PCR (qRT-PCR) analysis. Primers were designed using Primer3 (Version 4).^7^ NCBI Primer-BLAST^8^ was used for the specificity check of primers. The primer sequences are listed in Supplementary Table 1. Total RNA was isolated from germinating and non-germinating samples using the RNeasy Plant Mini Kit (Qiagen, Hilden, Germany) following the manufacturer’s protocol. To be used as an internal control, 200 μL of an overnight culture of the marine bacterium *Pseudoalteromonas prydzensis* Bowman 1998 was added to each sample before RNA extraction. The concentration of RNA was obtained using a Qubit^TM^ RNA BR Assay Kit (Invitrogen, Waltham, MA, United States). To remove any remaining genomic DNA from the samples, total RNA was treated with the DNase I (Qiagen, Hilden, Germany). DNase I-treated RNA was then subjected to cDNA synthesis using Superscript III reverse transcriptase (Invitrogen, Waltham, MA, United States). The amount of cDNA was measured with a NanoDrop^®^ ND-1000 spectrophotometer (ThermoFisher, Waltham, MA, United States). qPCR was performed in a Qiagen RotorGeneQ (Qiagen, Hilden, Germany). Real-time PCR reactions were prepared using iTaq Universal SYBR Green Supermix (Bio-Rad, NSW, Australia) in 20 μL volume. The qPCR conditions were 95°C for 2 min; 40 cycles at 95°C for 5 s, 59°C for 30 s. The RNA transcript level was presented as relative quantification calculated by the 2^–ΔΔ*CT*^ method (Livak and Schmittgen, 2001). The fold change of each gene was determined using three independent biological replicates.

### Statistical Analysis

The statistical procedures for qPCR experiments were performed using SPSS statistical software package version 24 (SPSS Inc, Chicago, IL, United States). One-way ANOVA was employed to calculate differences in the mean relative abundance of selected transcripts in germinating and non-germinating spores. A *P*-value <0.01 was considered statistically significant.

## Results

### RNA-Seq Strategy

For high-resolution capture of the transcriptional regulation of spore germination in *S. subterranea*, we used *de novo* assemblies from raw sequence data to infer transcript structures from the mapped reads in the absence of the annotated genome. The RNA extracted from the non-germinating and germinating spores of *S. subterranea* were sequenced at a depth of 100 million reads per sample and mapped to the available *S. subterranea* draft genome. A total of 380,678,646 reads were obtained, averaging 63,446,441 per sample and the majority of those reads from each library (84.42%) aligned to the *S. subterranea* reference genome (Table 1).

**TABLE 1:** Summary of RNA sequencing raw and alignment data in million reads from six RNA libraries of germinating and non-germinating spores of *S. subterranea*.

	**Replicate**	**Total reads**	**Alignment rate**
Germinating	1	65,676,382	84.52%
	2	55,385,215	84.93%
	3	66,197,900	86.12%
	**Mean**	**62,419,832**	**85.19% (53,175,454)**
Non-germinating	1	61,260,223	84.02%
	2	66,122,815	85.12%
	3	66,036,111	81.79%
	**Mean**	**64,473,050**	**83.64% (53,925,259)**
Total (Mean)		63,446,441	84.42%

### Global Transcriptional Changes in Response to Germination Stimulant

**FIGURE 1:**
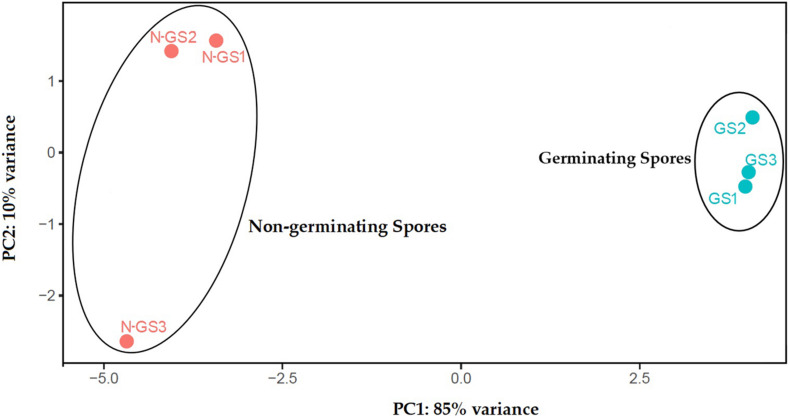
Principal component analysis (PCA) of transcriptome profiles based on normalised read counts per gene. RNA-seq analysis was performed with three biological replicates for germinating (GS) and non-germinating spores (N-GS).

After alignment to the *S. subterranea* genome and *de novo* reconstruction of the transcriptome, a total of 14,918 unigenes were identified which reduced to 6,664 after eliminating those with an average FPKM value less than 3. Of those, 5,448 unigenes (81.75%) were annotated with the non-redundant NCBI, UniProt, or String Protein databases with an E-value of 1 × 10^–5^. The remaining 1,216 unigenes (18.25%) were not annotated (Supplementary Table 2). The relationships between samples were studied by principal component analysis (PCA) which indicated that the biological replicates grouped closely together (Figure 1). PCA of the transcriptome data revealed the clear separation between germinating and non-germinating spores in PC1, which accounts for 85% of the variation between samples.

**FIGURE 2:**
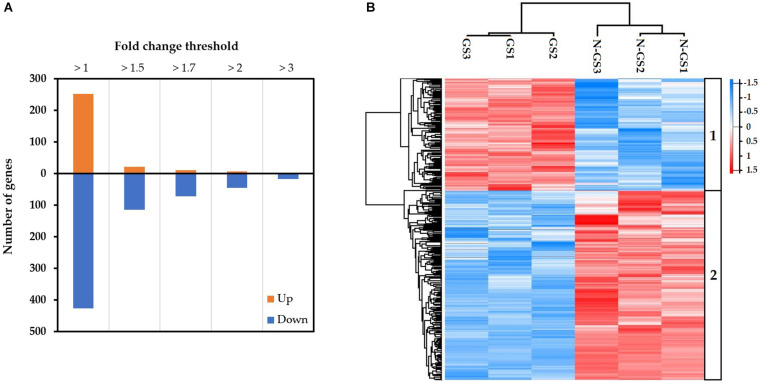
**(A)** The number of transcripts significantly (FDR < 0.05) differentially up or downregulated in the germinating vs non-germinating spores, expressed in fold changes. **(B)** Heatmap (based on Z-scores of the normalised expression of transcripts) showing the expression patterns of differentially expressed genes in response to germination stimulant.

The transcripts were further analysed to identify differentially expressed transcripts in germinating spores relative to non-germinating spores. A total of 679 differentially expressed transcripts were identified during the germination of *S. subterranea* resting spores (FDR < 0.05). Among these 252 were upregulated and 427 downregulated (Figure 2A). A Heatmap generated from the normalised expression of aligned reads of germinating and non-germinating spores (*n* = 3) revealed hierarchical clustering of all the differentially expressed transcripts with *P*-value < 0.05. The analysis yielded two major clusters of transcripts showing distinct patterns between germinating and non-germinating spores of *S. subterranea*. Cluster 1 contains transcripts with expression levels peaking in germinating spores (Figure 2B, top). Cluster 2 contains transcripts that had high expression levels in non-germinating spores, but low levels during germination (Figure 2B, bottom). A complete list of the annotated DEGs is provided as Supplementary Table 2.

### Gene Annotation and Differentially Expressed Gene Analysis

**FIGURE 3:**
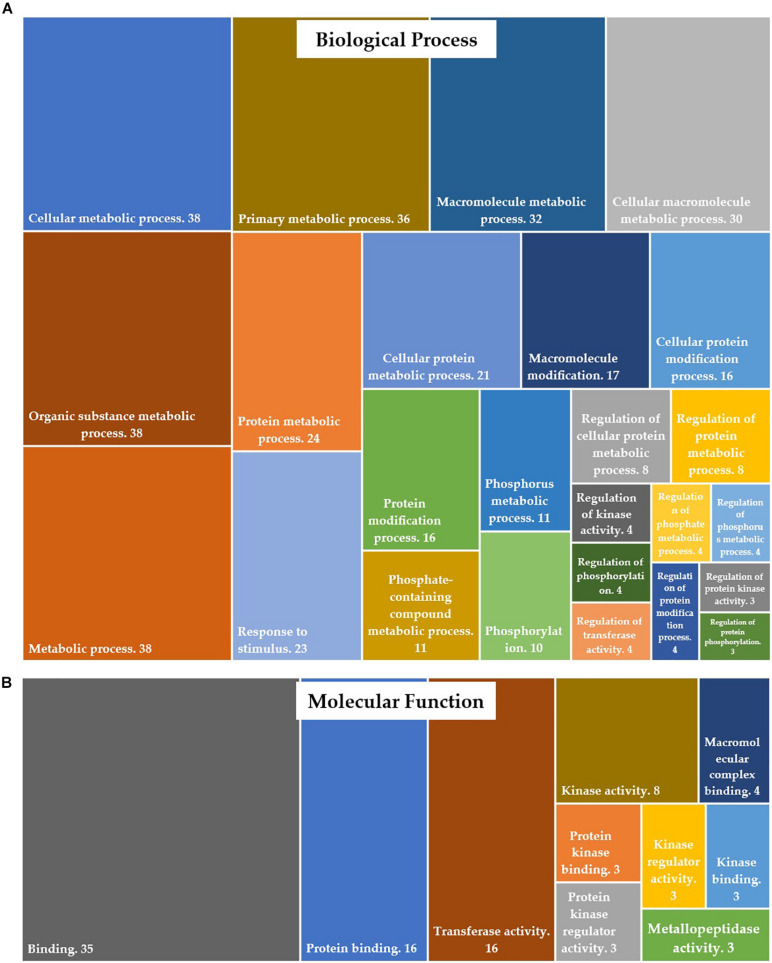
Distribution of DEGs into gene ontology (GO) categories, according to **(A)** their involvement in biological processes and **(B)** their molecular function.

Of 679 differentially expressed transcripts, 570 transcripts were annotated in protein databases. Gene ontology (GO) enrichment analysis of differentially expressed genes (DEGs) was conducted to identify the functional categories of the annotated genes. These DEGs were assigned to GO-terms for biological processes and molecular functions (David annotation). The significantly (*P*-value <0.05) enriched biological processes (top 25 only) and molecular functions are presented in Figure 3. The complete list of GO categories of DEGs is listed in Supplementary Table 3. The major functional categories of biological processes were as follows: cellular metabolic process, organic substance metabolic process and metabolic process (*n* = 38 each); primary metabolic process (*n* = 36); macromolecule metabolic process (*n* = 32); cellular macromolecule metabolic process (*n* = 30); protein metabolic process (*n* = 24); response to stimulus (*n* = 23) and cellular protein metabolic process (*n* = 21) (Figure 3A). Among the various categories, binding (*n* = 35), protein binding and transferase activity (*n* = 16 each) were dominantly represented within the molecular function category. Other significantly represented categories include kinase activity (*n* = 8) and macromolecular complex binding (*n* = 4) (Figure 3B).

**FIGURE 4:**
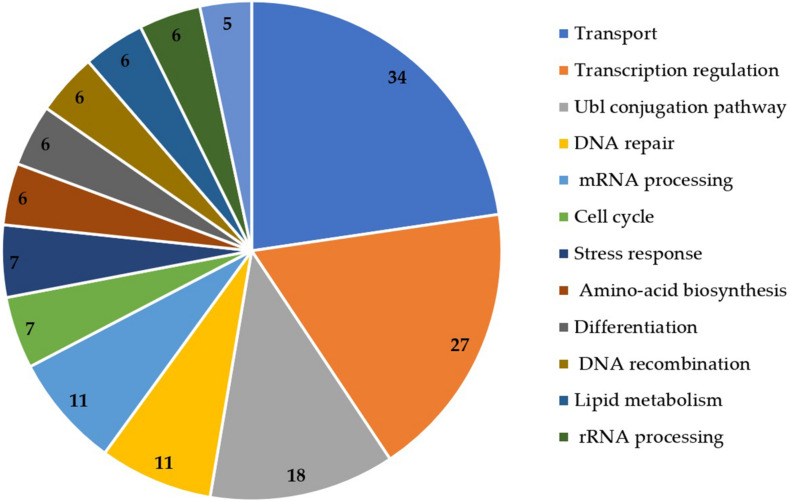
Functional annotation of DEGs obtained from UniProt database.

The functional annotation of DEGs was also retrieved from UniProt (Figure 4). Transport (*n* = 34), transcription regulation (*n* = 27), Ubl conjugation pathway (*n* = 18), DNA repair (*n* = 11) and mRNA processing (*n* = 11) were the most represented functions in the DEGs.

After obtaining the functional annotation of the identified transcripts from the gene and protein databases, we generated a list of selected DEGs with the possible role in the germination of resting spores in *S. subterranea* (Table 2). Among the upregulated group, the majority of genes were related to the initiation and regulation of transcription such as heat shock protein, transcription initiation factor and proteasome. The DNA repair genes such as DNA repair factor IIH helicase (Table 2 and Supplementary Table 2) were also found in the upregulated transcript. The multifunctional transcription factor, MADS−box transcription factor 6, was another upregulated gene. The expression levels of mannitol dehydrogenase, fatty acid synthase, trehalase and lipid droplet phospholipase which are involved in lipid and fatty acid metabolism were found to be downregulated upon germination. The mRNA expression of three important antioxidant genes was detected in the transcriptome of non-germinating and germinating spores of *S. subterranea*. While superoxide dismutase (SOD) was not found among the DEGs, the upregulation of glutathione S-transferase (GST) and the downregulation of catalase (CAT) was confirmed (Table 2 and Supplementary Table 2), highlighting the importance of these antioxidant enzymes in the protection of dormant and germinating spores from environmental stresses.

**TABLE 2:** DEGs with known role in the germination of resting spores.

**Transcript Id**	**Annotation Id**	**Description**
**Up**		
TCONS_00004168	Q9SSQ8	26.5 kDa heat shock protein, mitochondrial
TCONS_00010748	P22843	Histone H3
TCONS_00011859	A0A0H5RIM4	Chromo domain-containing protein
TCONS_00004586	Q9HFE8	Transcription-associated protein 1
TCONS_00013989	O00835	General transcription and DNA repair factor IIH helicase
TCONS_00000655	O43497	Voltage-dependent T-type calcium channel subunit alpha-1G
TCONS_00000548	Q67XX3	F-box protein
TCONS_00005155	F4JC97	Proteasome activator subunit 4
TCONS_00005901	P56839	Phosphoenolpyruvate phosphomutase (EC 5.4.2.9)
TCONS_00014747	A0A0H5RAN4	DUF4200 domain-containing protein
TCONS_00005328	Q6EU39	MADS-box transcription factor 6
TCONS_00003271	Q9ATB4	Transcriptional adapter
TCONS_00007933	Q9MA98	DNA excision repair protein ERCC-1
TCONS_00008936	P54211	Plasma membrane ATPase (EC 7.1.2.1) (Proton pump)
TCONS_00014207	A1Z9L3	ATP-dependent RNA helicase DHX8 (EC 3.6.4.13)
TCONS_00005711	Q10074	Vacuolar amino acid transporter 3
TCONS_00008752	Q43362	V-type proton ATPase 16 kDa proteolipid subunit
TCONS_00008729	Q54CN8	Transcription initiation factor TFIID subunit 13
TCONS_00005526	Q9FHE1	Glutathione S-transferase T3 (EC 2.5.1.18)
TCONS_00012137	P56282	DNA polymerase epsilon subunit
TCONS_00009388	A0A0H5R1C2	RNase H domain-containing protein
**Down**		
TCONS_00008644	Q9ZRF1	Probable mannitol dehydrogenase (EC 1.1.1.255)
TCONS_00010484	A0A0H5RB43	Zn (2)-C6 fungal-type domain-containing protein
TCONS_00011177	Q08448	Lipid droplet phospholipase 1 (EC 3.1.1.4)
TCONS_00009401	O93662	Catalase (EC 1.11.1.6)
TCONS_00008033	P43098	Fatty acid synthase subunit alpha (EC 2.3.1.86)
TCONS_00003907	P25867	Ubiquitin-conjugating enzyme E2-17 kDa (EC 2.3.2.23)
TCONS_00000069	A0A0H5R2B3	NDC10_II domain-containing protein
TCONS_00000821	A0A0H5R7J3	alpha-1,2-Mannosidase (EC 3.2.1.-)
TCONS_00008810	Q7ZYC4	Long-chain-fatty-acid–CoA ligase ACSBG2 (EC 6.2.1.3)
TCONS_00002913	Q557D2	Glucose-6-phosphate 1-dehydrogenase (EC 1.1.1.49)
TCONS_00002390	P16905	cAMP-dependent protein kinase type I regulatory subunit
TCONS_00011972	P71741	Trehalase (EC 3.2.1.28)
TCONS_00010927	Q02972	Cinnamyl alcohol dehydrogenase 8 (EC 1.1.1.195)
TCONS_00004841	A0A0H5QK57	DDE-1 domain-containing protein
TCONS_00000359	Q17551	E3 ubiquitin-protein ligase rpm-1 (EC 2.3.2.33)

*The annotation for unigenes was retrieved from the UniProt and NCBI databases using BLAST search.*

### Enzyme Classification

**FIGURE 5:**
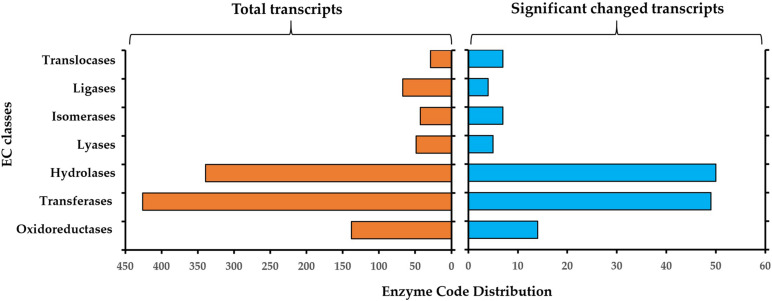
Enzyme classification (EC) codes for the total (left) and significantly changed transcript (right) upon *S. Subterranea* spore germination. Note that one transcript can be associated with more than one EC term.

The resulting enzyme classification (EC) for all 5448 annotated genes and for the significantly changed genes during germination is described in Figure 5. For all identified genes, the most representative enzyme classes were transferases (EC2, *n* = 426), hydrolases (EC3, *n* = 339) and oxidoreductases (EC1, *n* = 138). Other represented enzyme classes include ligases (EC6, *n* = 67), lyases (EC4, *n* = 49) and isomerases (EC5, *n* = 43) and translocases (EC7, *n* = 29) (Figure 4, left panel). In the significantly changed genes, the most representative enzyme classes were hydrolases (EC3, *n* = 50), transferases (EC2, *n* = 49) and oxidoreductases (EC1, *n* = 14) (Figure 5, right panel). Other represented enzyme classes include oxidoreductases (EC1), transferases (EC2) and isomerases (EC5). The higher abundance of the hydrolyses among the DEGs in comparison to the whole transcriptome indicated the important role of these enzymes in the transition from a dormant spore to a germinating spore in *S. subterranea*.

### KEGG Pathway Enrichment

KEGG pathway analysis of DEGs identified six significantly enriched metabolic pathways (FDR < 0.05) (Table 3). Some interesting pathways such as “arginine biosynthesis” and “biosynthesis of amino acids” which are involved in amino acid metabolism and might have a role in spore germination of *S. subterranea* identified in the DEGs. Arginine biosynthesis was considered to be involved in the pathogenesis of *Colletotrichum higginsianum* Saccardo (Takahara et al., 2012). A link between deficient amino acid biosynthesis and a loss, or reduction, in germination and pathogenicity has also been reported in several pathogenic fungi (Bailey et al., 2000; Solomon et al., 2000; Namiki et al., 2001).

**TABLE 3:** Pathway classification of differentially expressed genes (DEGs).

**Number**	**Pathway**	**False discovery rate**
1	Arginine biosynthesis	0.0263
2	2-Oxocarboxylic acid metabolism	0.0084
3	Nucleotide excision repair	0.0423
4	Spliceosome	0.0372
5	Biosynthesis of amino acids	0.048
6	Carbon metabolism	0.048

*Major pathway classes of DEGs (FDR < 0.05) were retrieved from KEGG and STRING databases.*

### Validation of RNA-Seq Data Using qRT-PCR

**FIGURE 6:**
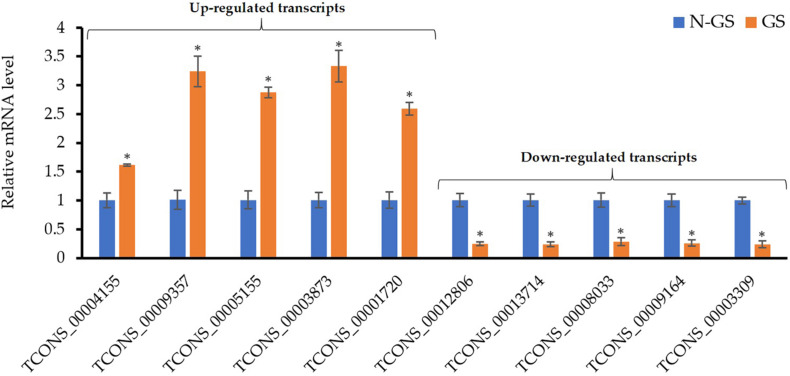
Validation of RNA-Seq results using qRT-PCR. Values are the means of three biological replicates ± SD. Asterisk (*) denotes a significant mRNA difference between germinating (GS) and non-germinating spore (N-GS) for each gene (*P*-value <0.01).

The expression levels of ten randomly selected genes from the DEGs were assessed using the qRT−PCR analysis to validate the RNA−seq experiment. Similar to the RNA-seq data, the relative expression analysis of the selected up (Figure 6, left) and downregulated transcripts (Figure 6, right) showed a significant (*P*-value <0.01) change in the mRNA level of these genes upon germination. The correlation between RNA-seq and qRT-PCR methods confirmed that the transcriptomics data are reliable.

## Discussion

The present study is the first to investigate the *in-vitro* germination process of *S. subterranea* by profiling the transcriptomics of germinating and dormant spores. The draft genome available for *S. subterranea* (Ciaghi et al., 2018) is not annotated and therefore information on the transcriptome of this pathogen is very limited. The presence of plant debris and rhizosphere microorganisms within the collected samples also complicate analyses. The establishment of the method for *in-vitro* germination of *S. subterranea* resting spores (Balendres et al., 2018) as well as the availability of the sporosori purification technique (Balotf et al., 2020), enabled us to efficiently analyse the transcriptome of the pathogen during the germination of resting spores. Our RNA-seq data was validated by gene expression analysis of randomly selected transcripts using qPCR analysis (Figure 6).

Germination of resting spores is highly regulated by metabolic processes and energy metabolism. It initiates a series of processes that gradually degrade the protective structures of the dormant spore and resume cellular processes (Moir and Cooper, 2016). In our experiment, 679 differentially expressed transcripts were identified during the germination of *S. subterranea* resting spores (Figure 2). The functional annotation and pathway analysis of DEGs demonstrated that most of these genes were involved in energy metabolic processes, transcription and translation, amino acid biosynthesis, transport, fatty acid metabolism, stress response, DNA repair, binding and transferase activity (Figures 3, 4 and Table 3). Similar results have been reported in the spore germination of other pathogens (Bindschedler et al., 2009; Ah-Fong et al., 2017; Bobek et al., 2017). Sharma et al. (2016) showed that the majority of changes in transcriptome during spore germination of *Fusarium oxysporum* Schlechtendal was related to energy metabolism functions including fatty acid biosynthesis, amino acid metabolism, purine metabolism and glycolysis. In *Penicillium digitatum* (Persoon) Saccardo, an elevated energy metabolism has been reported during spore germination with low concentration limonene treatment (Tao et al., 2019). Metabolomics analysis of *P. digitatum* revealed that central carbon and energy metabolism were upregulated during germination of spores (Che et al., 2020). In line with previously reported works on gene expression during spore germination (Luo et al., 2020; Swarge et al., 2020), important cellular metabolic pathways including fatty acid biosynthesis and amino acid biosynthesis showed significant changes in the germinating spores in comparison with dormant spores (Figure 4 and Table 3). In the lipid fraction, there is a decrease in fatty acid synthase and trehalase activity (Table 2). The germination of resting spores depends on several internal and external factors such as the composition of carbohydrates, mainly trehalose (Feofilova, 1992) and lipids (Mysyakina et al., 2018). In *Cunninghamella echinulata* (Thaxter) Thaxter (Mysyakina et al., 2016), *Saccharomyces cerevisiae* Meyen ex E.C. Hansen (Katohda et al., 1987), *Nosema algerae* Vávra & Undeen (Undeen et al., 1987) and *Umbelopsis ramanniana* (Möller) W. Gams (Mysyakina et al., 2016) the trehalose content of germinated spores decreased. Trehalose is a widespread molecule in nature and it has been established that it is a source of energy and is a reserve carbohydrate (Elbein et al., 2003). In *Streptomyces griseus* (Krainsky) Waksman & Henrici, glucose accumulation in spores caused an increased concentration of intracellular ATP and restored the activity of the trehalase enzyme (Mcbride and Ensign, 1987). Activated trehalase initiates the mobilisation of trehalose in swelled spores during early germination and ensures that spores are supplied with the necessary intermediates and energy (Thevelein et al., 1984; Chrungu et al., 2017). Due to the degradation of trehalose into higher concentrations of smaller molecules, the osmotic pressure increases until the filament begins its emergence and facilitates the germination processes (Undeen, 1990). Germination of resting spores might remove the need for storage carbohydrates such as mannitol and trehalose. Mannitol accumulation in the biotrophic plant pathogen *Uromyces fabae* (Persoon) Schröter resting spore is accompanied by transcript expression of a mannitol dehydrogenase. Kinetic analyses of the mannitol dehydrogenase indicate that the enzyme might be responsible for the utilisation of mannitol in spores (Voegele et al., 2005). The downregulation of lipid droplet phospholipase and mannitol dehydrogenase (Table 2) was also detected in our experiment which provides further evidence of the changes in energy metabolism during the germination of *S. subterranea* resting spores. Yang et al. (2017) observed that *Schizosaccharomyces pombe* Lindner spores lacking the ability to form lipid droplets failed to germinate. They concluded that lipid droplet dynamics is essential for spore survival. Taken together, changes in the composition and amounts of lipids and carbohydrates may provide the required energy for the transition from the dormant state to germination of *S. subterranea* resting spores.

Many genes involved in transcription and translation (e.g., heat shock proteins, histones and proteasome activator; Table 2 and Supplementary Table 2) were upregulated during the germination of resting spores (Figure 4). Similar results were found in the proteome analysis of the germinating and non-germinating spores of *S. subterranea* (Balotf et al., 2021). The end of spore dormancy is a process that needs the progressive re-starting of gene expression and protein synthesis processes. In *Neurospora crassa* Shear & B.O. Dodge, the most expressed genes during the early stage of conidial germination are heat shock proteins (Kasuga et al., 2005). These proteins have an important role in translation and are responsible for the correct folding of the newly synthesised proteins. We found five proteins from the heat shock proteins family in the DEGs (Supplementary Table 2) which is evidence of the dependence of spore germination on protein synthesis (González-Rodríguez et al., 2015). In addition, the upregulation of transcription-associated protein, histones and proteasomes was found in the transcriptome of germinating and non-germinating spores of *S. subterranea* (Table 2). These proteins are responsible for the initiation and control of transcription and were identified in the transcriptome and proteome of several bacterial and fungal pathogens during the germination of resting spores (Govin and Berger, 2009; Liu et al., 2016). The transcription activation could serve as biological switches functioning to control protein synthesis when exiting dormancy (Zhou et al., 2019).

In this study, we confirmed the mRNA expression of catalase (CAT), superoxide dismutase (SOD) and glutathione S-transferase (GST) in the transcriptome of *S. subterranea* resting spores (Table 2 and Supplementary Table 2). These are the key antioxidant enzymes participating in the ROS homeostasis of living organisms. Similar to our results here, the downregulation of CAT and upregulation of GST have been detected in the germination of resting spores in other pathogenic microorganisms. In *P. digitatum*, GST1 and CAT1 were downregulated, whereas GST2 and CAT2 were upregulated (Tao et al., 2019). Suo et al. (2015) found that the activities of GST and peroxidase (POD) were clearly induced during spore germination of *Osmunda cinnamomea* Linnaeus, while the CAT activity was decreased. The downregulation of CAT activity was also reported in the *Botrytis cinerea* Persoon during the germination of spores (González-Rodríguez et al., 2015). The expression levels of SOD did not change during *S. subterranea* spore germination (Supplementary Table 2). Superoxide dismutase is a metal-containing enzyme that catalyses the dismutation of superoxide to produce oxygen and hydrogen peroxide. It has been shown that SOD contributes to virulence in multiple pathogens including *Mycobacterium tuberculosis* (Piddington et al., 2001), *Puccinia triticina* (Wang et al., 2020) and *Rhizoctonia solani* Kühn (Foley et al., 2016). Cybulski et al. (2009) demonstrated the role of SOD in protecting *Bacillus anthracis* Cohn 1872 spores from oxidative stress. It has also been confirmed that the activity of SOD is essential for spore germination in *Schizosaccharomyces pombe* (Plante et al., 2017). Thus, the expression of SOD in both dormant and germinating phases might be related to its contribution to the protection of resting spores from environmental stresses and its role in the germination of spores. These reports, together with our results, indicated that antioxidant activity homeostasis is important in the maintenance of dormancy as well as in the germination of the pathogen. Several transcripts related to general DNA repair were also expressed actively during the germination of *S. subterranea* resting spores (Table 2 and Figure 4). The presence of “nucleotide excision repair” in the KEGG pathway enrichment (Table 3) emphasised the importance of DNA repair during the germination of the pathogen. Moeller et al. (2014) showed that *Bacillus subtilis* (Ehrenberg) Cohn spores deficient in DNA repair were more sensitive to ionizing radiation than wild-type spores. In dormant spores, DNA is believed to be in a supercoiled state, providing protection against damage. The rapid relaxation of the supercoiled DNA is necessary for an efficient reactivation of transcription during the early stages of germination (Setlow, 1995). Therefore, the upregulation of DNA repair genes such as DNA excision repair protein ERCC-1 or DNA repair factor IIH helicase subunit XPB (Table 2) in the transcriptome of *S. subterranea* during the germination of resting spores might accelerate the transcription process.

The presence of MADS−box transcription factor 6 in the DEGs was another interesting finding of the present study. The MADS-box family proteins are conserved in nearly all eukaryotes and play important roles in signal transduction, responses to environmental stresses and developmental control in plants, fungi and animals. Leesutthiphonchai and Judelson (2018) showed that a MADS−box transcription factor regulates a central step in sporulation of the oomycete *Phytophthora infestans* (Montagne) de Bary by regulating about 3000 sporulation−associated genes. Their results demonstrated that both mRNA and protein levels of MADS−box transcription factor decline upon *P. infestans* spore germination. They concluded that the expression of the MADS−box transcription factor is required for sporulation but not hyphal growth or plant colonisation (Leesutthiphonchai and Judelson, 2018). In contrast to this result, our transcriptome data showed the upregulation of MADS-box transcription factor 6 during the germination of *S. subterranea* resting spores (Table 2). In *B. cinerea*, the MADS-Box transcription factor Bcmads1 was required for growth, sclerotia production and pathogenicity of the pathogen (Zhang et al., 2016). Bcmads1 was also required for the full virulence potential of *B. cinerea* on apple fruit. Their results suggest that Bcmads1 may regulate pathogenicity by its effect on the protein secretion process. The MADS-box transcription factor was also involved in the cell wall synthesis, actin cytoskeleton organisation and spore coat stability in *Dictyostelium* sp. (Escalante et al., 2004). In the present study, the upregulation of the MADS-box transcription factor 6 was confirmed and our previous study demonstrated the decrease in actin protein during the germination of *S. subterranea* resting spores (Balotf et al., 2021). Therefore, we concluded that the presence of MADS-box transcription factor 6 in our RNA-seq data could be related to its role in the actin cytoskeleton organisation (Xiong et al., 2016).

We confirmed the presence of several enzymes in the transcriptome of *S. subterranea* (Figure 5). In the DEGs hydrolases were the most presented enzyme type showing the importance of these enzymes in the germination of *S. subterranea* resting spores. Hydrolases are needed for the initial reconstruction of the cell wall during the sporulation, spore maturation period and spore germination (Lim et al., 2001; Haiser et al., 2009). These enzymes facilitate the reconstruction of the cell wall allowing the entrance of external nutrients which is necessary for the initiation of germination. In *Streptomyces coelicolor* (Müller, 1908; Waksman and Henrici, 1948), mutants of two cell wall hydrolases showed slower germination (Haiser et al., 2009). These enzymes are known to participate in the growth restoration in *Streptomyces*, *Mycobacterium* and *Micrococcus* spp. (Mukamolova et al., 1998; Keep et al., 2006). Hydrolases were also shown to be important for the pathogenicity in *Colletotrichum lagenarium* Damm, P.F. Cannon & Crous (Takano et al., 1995). The mRNA expression of these enzymes during the germination of *S. subterranea* resting spores might therefore induce restoration of dormant spores by increasing the absorption of nutrients (Bobek et al., 2017). Using GO enrichment analysis of DEGs, we have also identified several molecular functions related to phosphorylation and kinase activity (Figure 3B). The protein kinases are a large superfamily of enzymes that catalyse the modification of proteins by phosphorylation (Manning et al., 2002). Nguyen et al. (2016) investigated the role of protein kinases in the resting spore germination in *Bacillus subtilis*. The results of their study showed that the phosphorylation of the spore coat protein H, which is a protein kinase, was required for the efficient germination of *B. subtilis* spores. The role of cAMP/protein kinase A signaling in the germination of *Schizosaccharomyces pombe* has also been confirmed (Hatanaka and Shimoda, 2001). In the soilborne plant pathogen *Verticillium dahlia* Klebahn, the mutant for cAMP-dependent protein kinase had similar growth rates to those of wild-type strains, while conidia production was significantly reduced and spore germination was slightly increased suggesting the inhibitory role of this protein kinase in the germination of spores (Tzima et al., 2010). Consistent with this result, the mRNA level of cAMP-dependent protein kinase decreased in the germinating spores in comparison to non-germinating spores of *S. subterranea* (Table 2). Although not enough is known about the signaling pathway in the germination processes of plasmodiophorids, our transcriptome data suggesting the importance of kinase activity and cAMP-dependent protein kinase in the germination of dormant spores in *S. subterranea*.

One of the major significantly over-represented group of functional genes identified in DEGs were genes involved in the transport of various molecules such as amino acid, protein and ion transport (Figure 4 and Table 2). The activities of these transporters are considered essential for the successful germination of dormant spores (Dembek et al., 2013). The roles of the amino acid and protein transporters in the germination processes of *S. subterranea* are unknown at present. The involvement of ions during the germination process has been studied before and a potential explanation for the upregulation of calcium channel during the germination of dormant spores (Table 2) is that divalent cations such as calcium, zinc and manganese accumulate in resting spores during spore formation (Charney et al., 1951) and rapid release of these cations is distinct early germination (Swerdlow et al., 1981).

In conclusion, the results of the present study provide a comprehensive overview of the changes in transcriptome during the germination of *S. subterranea* resting spores and extend our knowledge on the spore germination in plasmodiophorids. Employing a *de novo* reconstruction of the spore’s transcriptome, we identified several genes with a potential role in the spore germination and pave the way for future studies on the molecular mechanisms of the germination in the complex obligate biotrophic soilborne pathogens.

## Data Availability Statement

The data presented in this study are deposited in the NCBI database (https://ncbi.nlm.nih.gov), accession number PRJNA720224.

## Author Contributions

CW, SB, RT, and DN designed the experiments. SB performed the experiments, analysed the data, and prepared the original draft. CW, RT, and DN reviewed and edited the manuscript. CW and DN financially supported the project. All authors provided critical feedback to the article and approved the submitted version.

## Conflict of Interest

The authors declare that the research was conducted in the absence of any commercial or financial relationships that could be construed as a potential conflict of interest.

## Footnotes

1 https://www.uniprot.org/

2 https://david.ncifcrf.gov/

3 www.genome.jp/kegg/

4 https://string-db.org/cgi/input

5 https://enzyme.expasy.org/

6 www.perseus-framework.org

7 www.bioinfo.ut.ee/primer3-0.4.0/

8 https://www.ncbi.nlm.nih.gov/tools/primer-blast
