# Supplementary material for: Spore Germination of the Obligate Biotroph Spongospora subterranea: Transcriptome Analysis Reveals Germination Associated Genes
Source: Front Microbiol. 2021 Jun 16;12:691877. doi: 10.3389/fmicb.2021.691877 (PMC8256667; doi:10.3389/fmicb.2021.691877)
Supplement: Supplementary file 4 [file Data_Sheet_1.PDF]

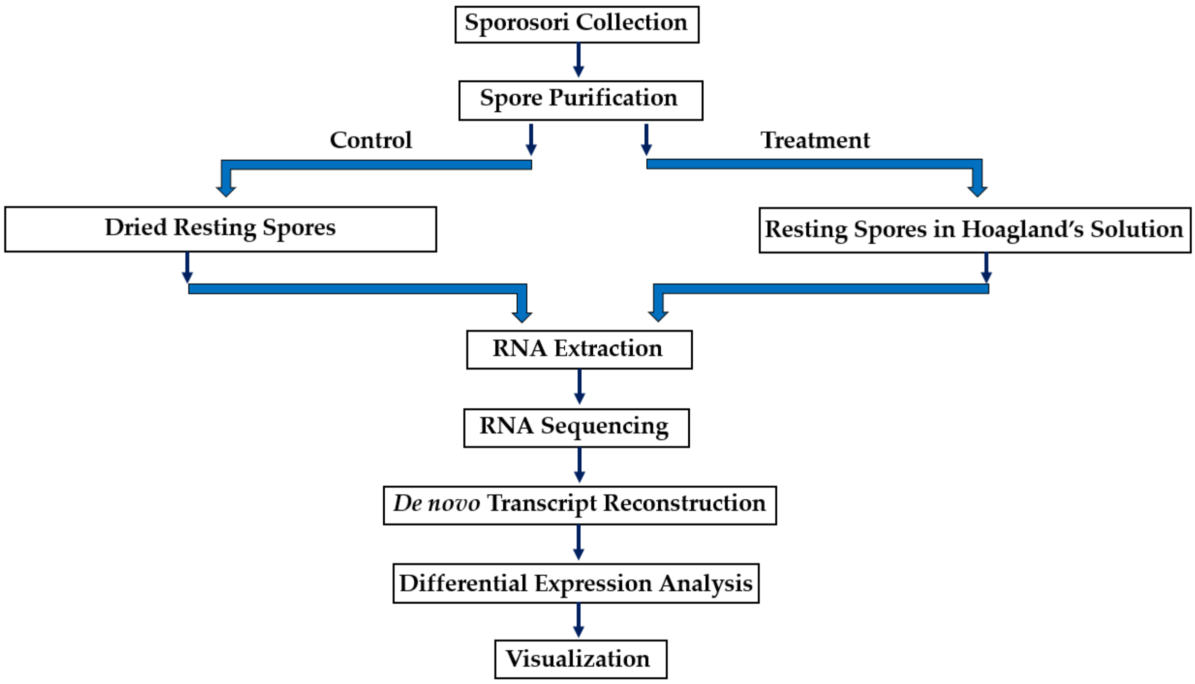

Supplementary Figure 1. Workflow for the RNA-seq experiments. *Spongospora subterranea* sporosori was collected from powdery scab-infected potato tubers and purified using Ludox® centrifugation. The purified spores were divided into two parts: treatment and control. In the treatment group, resting spores were suspended in Hoagland's solution. Dried resting spores served as an ungerminated control. RNA was extracted from all samples and sequenced on a NovaSeq 6000 instrument. RNA-seq raw data were processed using the *de novo* transcript reconstruction protocol. Galaxy platform and Perseus software were used for the visualization of data.
